# BAMBI regulates macrophages inducing the differentiation of Treg through the TGF-β pathway in chronic obstructive pulmonary disease

**DOI:** 10.1186/s12931-019-0988-z

**Published:** 2019-02-06

**Authors:** Sheng-Wen Sun, Long Chen, Mei Zhou, Jiang-Hua Wu, Zhao-Ji Meng, Hong-Li Han, Shuai-Ying Miao, Chen-Chen Zhu, Xian-Zhi Xiong

**Affiliations:** Department of Respiratory Medicine, Union Hospital, Tongji Medical College, Huazhong University of Science and Technology, 1277 Jiefang Avenue, Wuhan, 430022 China

**Keywords:** COPD, M2 macrophages, Regulatory T lymphocytes, TGF-β/Smad signalling pathway, BAMBI

## Abstract

**Background:**

Chronic obstructive pulmonary disease (COPD) is characterized by continuous flow limitation and the immune system including macrophages and regulatory T lymphocytes (Tregs) is involved in COPD pathogenesis. In our previous study, we investigated that TGF-β/BAMBI pathway was associated with COPD by regulating the balance of Th17/Treg. However, the role of bone morphogenetic protein and activin membrane-bound inhibitor (BAMBI), a pseudoreceptor of TGF-β signalling pathway, in regulating the immune system of COPD patients has not been fully studied. Hence, we speculate that the pseudoreceptor BAMBI may play roles in the regulation of M2 macrophages to induce the differentiation of CD4+ naïve T cells into Tregs and influence the immune response in COPD.

**Methods:**

Peripheral blood mononuclear cells (PBMCs) were isolated from healthy nonsmokers (*n* = 12), healthy smokers (*n* = 10) and COPD patients (*n* = 20). Naïve CD4+ T cells and monocytes-induced macrophages were used for coculture assays. The phenotypic characteristics of macrophages and Tregs were determined by flow cytometry. The expression levels of BAMBI and the TGF-β/Smad pathway members in M2 macrophages were measured by a Western blot analysis. The monocyte-derived macrophages were stimulated with cigarette smoke extract (CSE, concentration of 0.02%) to simulate the smoking process in humans. pCMV-BAMBI was transfected into monocyte-derived M2 macrophages for subsequent co-culture assays and signalling pathway analysis.

**Results:**

Our results showed that M2 macrophages could induce the differentiation of Tregs through the TGF-β/Smad signalling pathway. In addition, monocyte-derived macrophages from COPD patients highly expressed BAMBI, and had a low capacity to induce Tregs differentiation. The expression of BAMBI and the forced expiratory volume in 1 second (FEV1%) were negatively correlated in COPD. Furthermore, overexpression of BAMBI promoted the conversion of M2 macrophages to M1 macrophages via the TGF-β/Smad pathway.

**Conclusions:**

We demonstrated that BAMBI could promote the polarization process of M2 macrophages to M1 macrophages via the TGF-β/Smad signalling pathway and that overexpression of BAMBI could decrease the ability of M2 macrophages to induce Treg differentiation. These findings may provide a potential mechanism by which blocking BAMBI could improve immune function to regulate COPD inflammatory conditions.

## Background

Chronic obstructive pulmonary disease (COPD) is a multifactorial pulmonary disorder characterized by not fully reversible airflow obstruction [1, 2], which results from the abnormal airway inflammation induced by inhaled particles and noxious gas [3]. COPD is a major public health issue and will become the third leading cause of death worldwide by 2020 [4];the increased morbidity and mortality of COPD result in heavy burdens [5]. Cigarette smoke is an important risk factor for COPD [6]. The imbalance of inflammatory and anti-inflammatory responses largely accounts for COPD [7], and macrophages and regulatory T lymphocytes (Tregs) are proposed to be the main orchestrators of the chronic inflammatory responses [8].

Tregs belong to a CD4+ T cell subgroup functioning as a modulatory of immune response and can be divided into natural regulatory T cells (nTregs) and induced regulatory T cells (iTregs) [9, 10]. The transcription factor forehead Box P3 (Foxp3) is considered a specific marker of Tregs [11]. The number of Tregs in peripheral blood from COPD patients was lower in healthy individuals, and compared to healthy controls, these cells had a lower capacity to regulate the pro-inflammatory and anti-inflammatory balance in the COPD patients [12].

Generally, macrophages consist of two polarization states, M1 (classically activated macrophages) and M2 (alternatively activated macrophages). Functionally, M1 macrophages represent pro-inflammatory macrophages; express HLA-DR, CD40, CD80 and CD86; secrete many proinflammatory cytokines (IL-12, TNF-alpha, IFN-γ, IL-1β and IL-6) [13]. M2 polarized macrophages are anti-inflammatory macrophages that express the scavenger receptor (CD163) and the mannose receptor (CD206) [14]; M2 macrophages are highly phagocytic and secrete high levels of IL-10 and transforming growth factor β1 (TGF-β1) [15]. In general, M1 macrophages promote Th1 responses, while M2 macrophages drive Th2 responses [16]. M2 macrophages can be divided into three subsets, namely, M2a, M2b and M2c [17]; among these subsets, M2a is induced by IL-4 and IL-13, M2b is induced by immunocomplexes and LPS and M2c is induced by IL-10, TGF-β and glucocorticoids [18].

As an important signalling pathway for inflammatory regulation, the TGF-β superfamily regulates cell growth, differentiation, migration and apoptosis in various cell types [19]. Generally, the TGF-β superfamily binds to the type I/II receptor and activates the signalling pathway by inducing the phosphorylation of downstream regulatory proteins of the SMAD family (R-SMADs) [20]. Bone morphogenetic protein and activin membrane-bound inhibitor (BAMBI) is a transmembrane glycoprotein that has a similar extracellular ligand binding domain structure as TGFβR1 but lacks an intracellular serine/threonine kinase domain [21]. Thus, BAMBI is believed to be a pseudoreceptor of the TGF-β signal pathway and may have a negatively regulatory role in the TGF-β signalling pathway [22]. In fact, BAMBI has been identified as an inhibitor of TGF-β signalling and is regulated by the Wnt/β-catenin pathway [23]. In our previous study, we found that BAMBI expression in the peripheral blood lymphocytes in COPD patients was increased [24], and BAMBI levels were shown to be markedly increased in alveolar macrophages from COPD patients [25].

Although BAMBI has been shown to play critical roles in several physiological processes, the immunological features of BAMBI in monocyte-derived macrophages from COPD patients have not been reported, and whether BAMBI can regulate the phenotype and function of macrophage and the potential signalling pathways remain unknown. In addition, M2 macrophages have been reported to induce regulatory T cells via membrane-bound TGF-β1 [25], but the exact mechanism is not clear. Hence, we speculate that the pseudoreceptor BAMBI may play roles in the regulation of M2 macrophages inducing Tregs and influence the immune response in COPD.

In the current study, we first determined whether monocyte-derived M2 macrophages could induce the differentiation of CD4+ T cells into regulatory T cells and then studied the effects of CSE on M0 macrophages. We further investigated the expression of the TGF-β signalling pathway and BAMBI in healthy controls (HC), healthy smokers (HS) and COPD patients. Finally, we explored the effects of upregulating BAMBI in monocyte-derived macrophages and the potential mechanisms involved.

## Methods

### Subjects and sample collection

A total of 42 subjects were recruited, including 12 healthy nonsmoker controls (HC group), 10 healthy smokers (HS group) and 20 stable COPD patients (COPD group); the COPD patients (11 patients with mild to moderate COPD and nine with severe and very severe COPD) were included based on the criteria of the Global Initiative for Chronic Obstructive Lung Disease (GOLD) guidelines. The mild to moderate COPD patients were treated with long-acting β2 agonists (LABA), while the severe and very severe COPD patients with LABA combined with inhaled glucocorticoids. All patients were free of exacerbation for at least 4 weeks before the study. The detailed clinical information for each group is provided in Table 1. Our study was approved by the Ethics Committee of Union Hospital, Tongji Medical School, Huazhong University of Science and Technology (#2013/S048), and the donors provided informed and signed consent allowing their samples to be used for scientific purposes.

**Table 1:** The basic clinical characteristics of the participants in different groups

Variables	HC (*n* = 12)	HS (*n* = 10)	COPD (*n* = 20)
Age, years	52.1 ± 1.0	55.5 ± 2.0	59.1 ± 1.7
Gender (male/female)	10/2	10/0	19/1
Smoking (pack-year)	–	40.8 ± 3.4	42.5 ± 5.5
FEV1(% predicted)	103.1 ± 1.5	98.7 ± 2.9	62.1 ± 5.5 ^***,#*##*^
FEV1/FVC (%)	84.0 ± 0.7	83.4 ± 0.1	54.0 ± 2.7 ^***,#*##*^
White blood cell (× 10^9^/L)	6.1 ± 0.4	6.4 ± 0.4	6.6 ± 0.3

The data are represented as the mean ± SEM. *HC* healthy controls, *HS* healthy smokers, *COPD* chronic obstructive pulmonary disease, *FEV1* forced expiratory volume in 1 second, *FVC* forced vital capacity, *WBC* white blood cell. ^***^P < 0.001 vs. the HC group, ^#*##*^*P* < 0.001 vs. the HS group

### Cell purification and culture

Peripheral blood mononuclear cells (PBMCs) were isolated from fresh peripheral blood using Lymphocyte Separation Medium (MP Biomedicals, Illkirch, France). CD4+ naïve T cells (naïve CD4+ T Cell Isolation Kit II, Miltenyi Biotec, Germany) and CD14+ monocytes (Monocyte Isolation Kit II, Miltenyi Biotec) were isolated from PBMCs by magnetic activated cell sorting (Miltenyi Biotec) according to the manufacturer’s instructions. The purity of CD4+ naïve T cells and CD14+ monocytes was > 97% as measured by flow cytometry. All cells were cultured in complete RPMI 1640 medium (Gibco, Grand Island, NY, USA) containing 10% foetal bovine serum (FBS) and placed in an incubator at 37 °C in 5% CO_2_.

### Macrophage subset generation

Macrophage subtypes were generated by addition of relevant exogenous cytokines. The M1 phenotype was generated by exposing monocytes to 50 ng/ml Granulocyte-Macrophage Colony Stimulating Factor (GM-CSF) for 6 days and 20 ng/ml IFN-r and 20 ng/ml LPS for an additional 24 h. To generate the M2 phenotype, we first exposed monocytes to 50 ng/ml Macrophage Colony Stimulating Factor (M-CSF) for 6 days and then 20 ng/ml IL-4 and 20 ng/ml IL-13 for 24 h.

### Coculture assays

The coculture assays were performed by the addition of 2 × 10^5^ M1 or M2 or BAMBI-upregulated macrophages (designated BUM) to 4 × 10^5^ CD4+ naïve T cells with IL-2 (50 ng/ml) after anti-CD3 and anti-CD28 stimulation for 72 h. To determine whether the TGF-β signalling pathway was involved in the induction process, we added LY2109761 (10 μg/ml, Selleckchem, USA), an inhibitor of TGF-β receptor kinase, to the mixed cocultures. The cell supernatants before or after coculture of M2 macrophages were collected for enzyme-linked immunosorbent assays (ELISAs).

### ELISAs

The concentrations of IL-10 (Booster, Wuhan, China), IL-12p70 (Booster), TNF-a (Neobioscience, Shenzhen, China) and TGF-β1 (Neobioscience) in culture supernatants were analysed by ELISA kits according to the manufacturers’ instructions. The plates were read at a wavelength of 450 nm.

### Flow cytometric analysis of cell surface markers and intracellular staining

The expression of surface markers and intracellular staining were assessed by flow cytometry. The following fluorescence-coupled antibodies were used in the study: fluorescein isothiocyanate (FITC)-conjugated anti-CD4 (eBioscience, San Diego, CA, USA), FITC-conjugated anti-CD8 (BD Biosciences, San Jose, CA, USA), phycoerythrin (PE)-Cy7-conjugated anti-CD25 (BD Biosciences), and PE-conjugated anti-Foxp3 (eBioscience). APC-Cy7-conjugated anti-CD14 (BD Biosciences), BV510-conjugated anti-CD86 (BD Biosciences), PE-conjugated anti-CD163 (BD Biosciences), and FITC-conjugated anti-BAMBI (eBioscience) were also used. The relevant isotype controls were applied to confirm specific binding. Flow cytometry was performed on a BD LSRFortessa X-20 and analysed using FlowJo V10 software. CD14, CD86, and CD163 were assessed to validate the phenotypes of the macrophages; CD14 indicates the whole macrophage, CD86 indicates the M1 phenotype, and CD163 indicates the M2 phenotype. As previously described, CD4+ CD25+ FoxP3+ T cells represent Tregs.

### Transfection

The BAMBI plasmid (0.8 μg) or a control plasmid (0.8 μg) was transfected into monocyte-derived macrophages (5 × 10^5^ cells) from healthy subjects at a confluence of 90% in a 24-well plate with Lipofectamine 3000 following the manufacturer’s protocol (Invitrogen). Cells were harvested for the subsequent experiments after 48 h of transfection. The verification of BAMBI overexpression was performed by real-time PCR (RT-PCR) and Western blotting before the subsequent analysis.

### Preparation of cigarette smoke extract (CSE)

The CSE was prepared using our previous method described by Blue and Janoff [26]. Briefly, cigarette smoke was drawn into a 15 ml plastic syringe, and then smoke was slowly bubbled into a tube containing 2.5 ml of RPMI 1640 medium with our specific device. A concentration of 100% CSE was produced from one cigarette (Huang Helou, Wuhan, Hubei, China). Next, the CSE solution was filtered via 0.22-μm filters. To ensure the activity of the substances in the CSE, we freshly prepared the solution for each experiment. To determine the appropriate concentration, we performed Cell Counting Kit-8 (CCK-8; Dojindo Laboratories, Tokyo, Japan) assays to detect cell cytotoxicity according to the manufacturer’s protocol. Approximately 5 × 103 cells were seeded onto 96-well plates, followed by pretreatment with a gradient concentration of 1, 0.05, 0.02, 0.002, and 0% CSE. Cells were then incubated with CCK-8 solutions for 2 h at 37 °C. The absorbance was measured at 450 nm using a SpectraMax M5 multifunctional microplate reader (Molecular Devises, Sunnyvale, CA, USA) at 30 min, 60 min, and 2 h. The cells used for the flow cytometry and PCR assays were isolated on the seventh days after the CSE stimulation.

### Western blot (WB) analysis

The macrophages were lysed in RIPA (Biosharp, Jiangsu, China) supplemented with 1 mM phenylmethanesulfonyl fluoride (PMSF). A total of 30 μg protein was separated by SDS-PAGE and then transferred onto polyvinylidene fluoride membranes (Millipore, Billerica, MA, USA). The membranes were blocked in TBST (TBS with 0.1% Tween 20) containing 5% nonfat milk for 1 h at room temperature and then incubated with primary antibodies: anti-BAMBI (R&D Systems, Minneapolis, USA), anti-TGF-β RI (Abcam, Cambridge, UK), anti-TGF-β RII (Abcam), anti-Smad2/3 (Cell Signaling Technology, USA), anti-phospho-Smad2/3 (Cell Signaling Technology), and anti-GAPDH (Cell Signaling Technology) antibodies, followed by the corresponding HRP-conjugated secondary antibodies (1:2000 dilution). All primary antibodies were used at a dilution of 1:1000. The membranes were washed 3 times in PBST and subsequently exposed to an enhanced ECL chemiluminescent substrate (Beyotime Biotech, Shanghai, China). GAPDH was used as the endogenous reference.

### RNA extraction, reverse transcription, and real-time PCR

Gene expression was quantified by qRT-PCR as described previously. Briefly, the total RNA was extracted by TRIzol reagent (TaKaRa, Dalian, China) according to the manufacturer’s protocols and the 260/280 ratio was detected by a NanoDrop ND-1000. Total RNA (500 ng) was reverse transcribed to cDNA using a PrimeScript RT reagent kit (TaKaRa). Subsequently, PCR amplification was measured with a StepOnePlus Real-Time PCR System (Applied Biosystems, Foster City, USA). The relative fold changes in gene expression were normalized to GADPH using the ΔΔCt method. The following primers were used:

Human BAMBI: forward 5′-catacccacattggaatgctgtc-3’

reverse 5′-tgcaccttggtgataaggtttctg-3’

Human CD163: forward 5′-gctacatggcggtggagacaa-3’

reverse 5′-atgatgagaggcagcaagatgg-3’

Human CD86: forward 5′-tggcctagggtacaggcaaca-3’

reverse 5′-gcccagatagaagtggctccag-3’

Human GAPDH: forward 5′-gcaccgtcaaggctgagaac-3’

reverse 5′-tggtgaagacgccagtgga-3’

### Statistical analysis

All results are presented as the mean ± standard deviation (SD). Unpaired student’s t-test was used for the analysis between two groups, and one-way analysis of variance (ANOVA) followed by Bonferroni’s multiple comparisons test was used for multiple comparisons. GraphPad Prism 6 software (GraphPad Software, La Jolla, USA) was used for data analysis and *P* < 0.05 was considered statistically significant. All experiments were repeated at least three times.

## Results

### M2 macrophages induced the differentiation of Tregs via the TGF-β/Smad signalling pathway

**Fig. 1:**
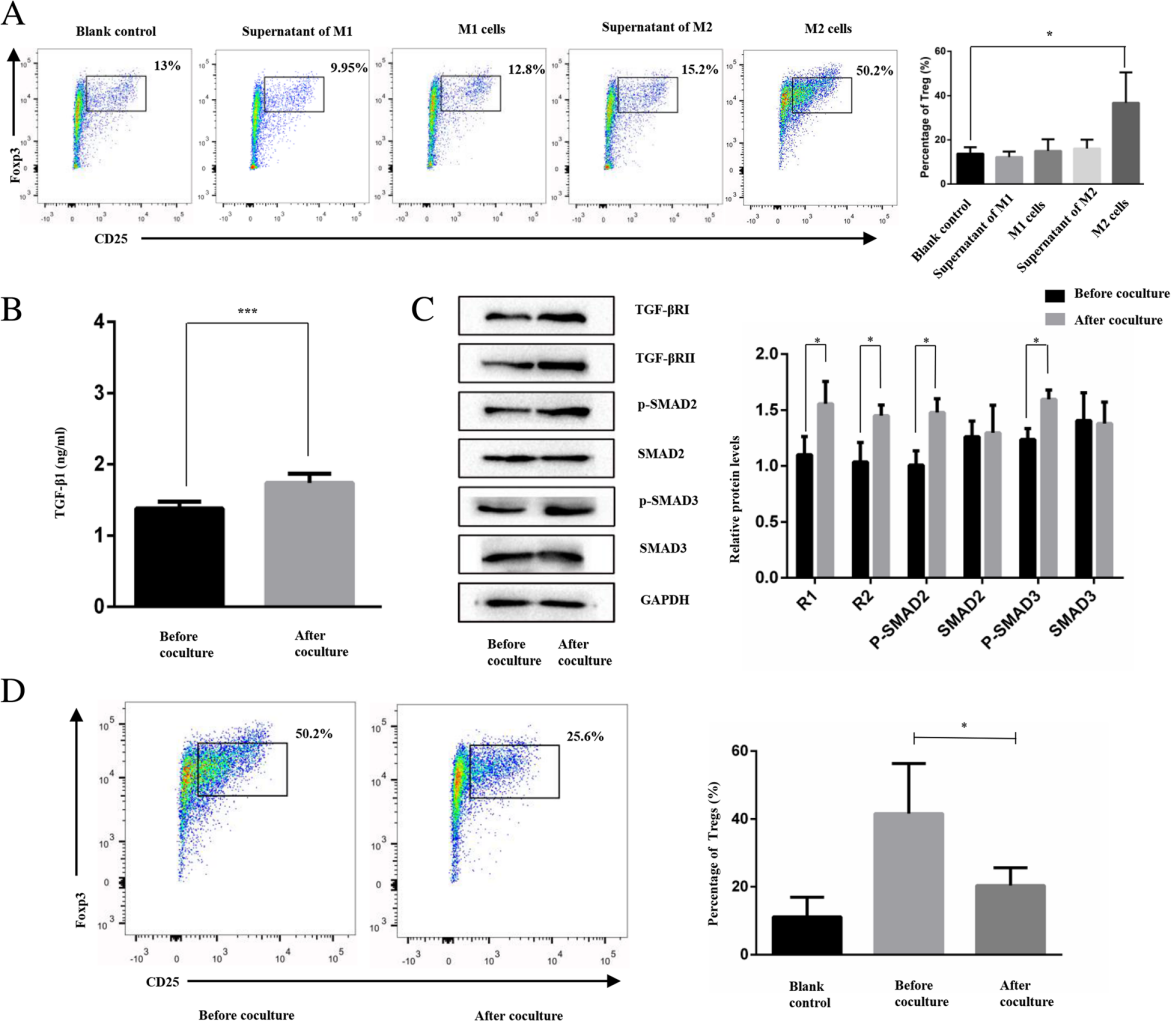
M2 macrophages induce the differentiation of CD4 + CD25 + Foxp3+ T cells (Tregs) via the TGF-β/Smad signalling pathway. **a**. Percentage of Tregs in different groups (blank control, supernatant of M1 cells, M1 cells, supernatant of M2 cells and M2 cells). **b**. Concentration of TGF-β1 in monocyte-derived M2 macrophages before and after coculture. **c**. Western blot analysis of TGF-β/Smad signalling pathway in monocyte-derived M2 macrophages. **d**. The proportion of Tregs decreased compared with that in the untreated group after addition of LY2109761. *P < 0.05, ***P < 0.001

To determine whether M2 macrophages induce the differentiation of CD4+ T cells into regulatory T cells, we cocultured naïve CD4+ T cells and macrophages or supernatant of macrophages for 3 days. As shown in Fig. 1a, the M2 macrophages promoted the proportion of CD4 + CD25 + FOX3+ Tregs (*P* < 0.05); however, the M1 cells and the supernatants of M1 or M2 macrophages did not show the capability of induction. The data presented here indicated that M2 macrophages, rather than M1 cells, induced the differentiation of Tregs.

As membrane-bound TGF-β1 was shown to participate in M2 macrophage-induced Treg differentiation [27], to examine whether the downstream TGF-β signalling pathway participated in this process, we isolated M2 macrophages and supernatant from the cocultures. As shown in Fig. 1b, the cell supernatant after coculture showed a higher concentration of TGF-β1 than that before the coculture. The difference was statistically significant (*P* < 0.01).

The WB results demonstrated that the protein levels of TGF-βRI/RII and p-SMAD2/3 in M2 macrophages were increased after the coculture compared to those before the coculture, but SMAD2/3 did not show a corresponding change (Fig. 1c).

Moreover, as shown in Fig. 1d, after the addition of LY2109761, which is an inhibitor of the TGF-β/Smad pathway, the proportion of Tregs was decreased compared with that in the untreated cells (*P* < 0.05).

These results indicate that the TGF-β/SMAD signalling pathway was involved in the process of M2 macrophage-induced Treg differentiation.

### CSE promoted the conversion of M0 to M2

**Fig. 2:**
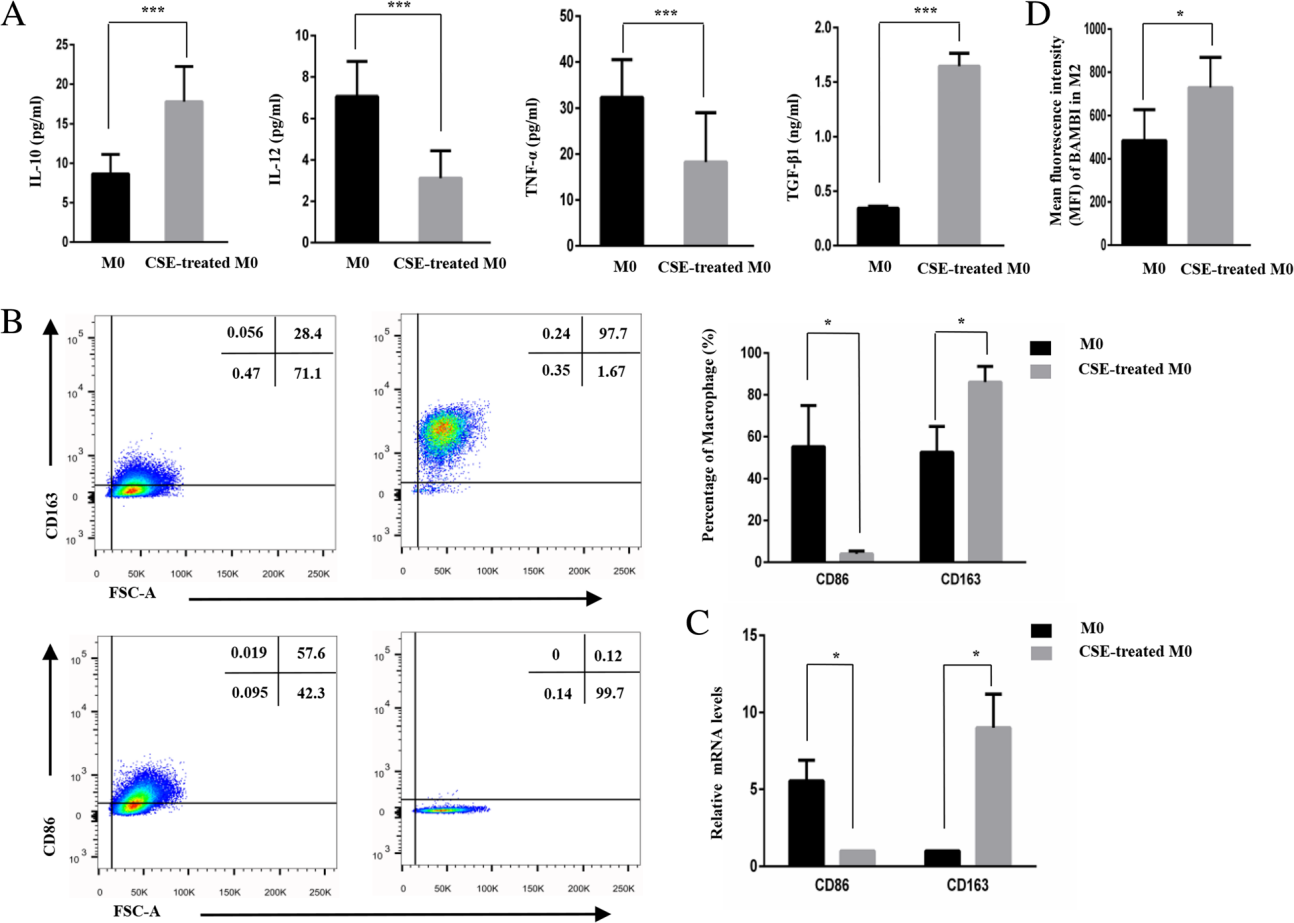
Cigarette smoke extract (CSE) promotes the conversion of M0 to M2 cells. **a**. Concentration of IL-10, IL12, TNF-α and TGF-β1 in the M0 group and CSE-treated M0 group. **b**. Expression levels of CD86 and CD163 in the CSE-treated group and M0 group as detected by flow cytometry assays. **c**. PCR assays showing that the CSE-treated M0 group had a lower CD86 level and a higher CD163 level than M0 group. **d**. The mean fluorescence intensity (MFI) of BAMBI in the CSE-treated macrophages and M0 macrophages. *P < 0.05, ***P < 0.001

To simulate the smoking process in vivo, we added CSE to the macrophages previously cultured in RPMI 1640 supplemented with 10% FBS and M-CSF (50 ng/ml). As shown in Fig. 2a, we found a higher secretion of the immunosuppressive cytokines IL-10 (*P* < 0.001) and TGF-β1 (*P* < 0.001) and lower levels of the pro-inflammatory cytokines IL-12 (*P* < 0.001) and TNF-α (*P* < 0.001) in CSE-treated macrophages than inM0 macrophages. Furthermore, as shown in Fig. 2b, the flow cytometry results confirmed that CSE-treated M0 cells had higher CD163 expression and lower CD86 expression than the untreated cells (*P* < 0.05). The corresponding PCR experiment (Fig. 2c) also verified these trends (*P* < 0.05). Figure 2d showed that the expression of BAMBI in CSE-treated macrophages was increased.

In conclusion, our study indicates that CSE-induced M0 cells exhibit the characteristics of M2 macrophages.

### TGF-β signalling pathway and BAMBI expression and inducibility of monocyte-derived M2 macrophages in COPD patients

**Fig. 3:**
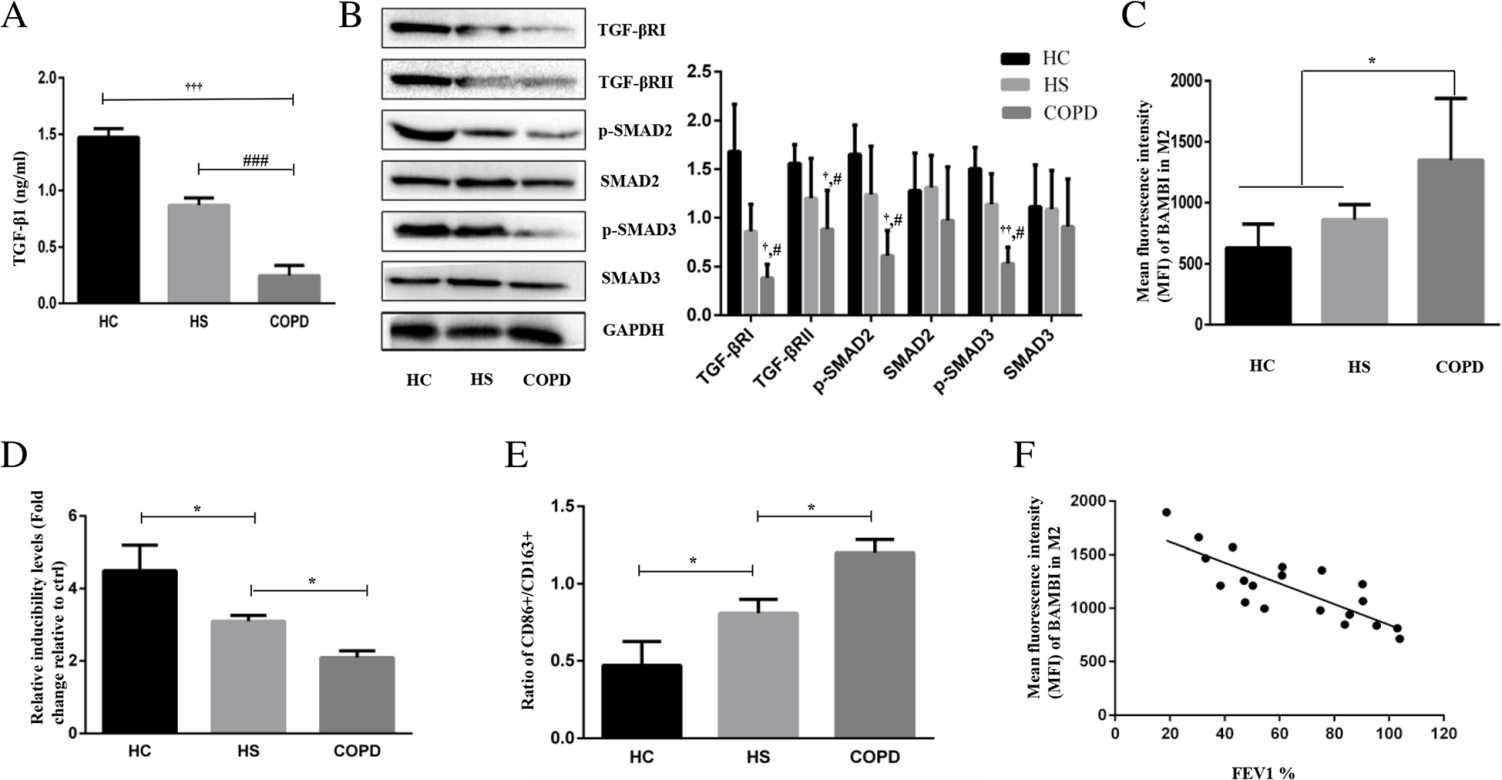
TGF-β signalling pathway and BAMBI expression and inducibility of monocyte-derived M2 macrophages in COPD patients. **a**. The concentration of the TGF-β1 in the HC, HS and COPD groups. **b**. WB analysis of TGF-βRI/II, p-SMAD2/3 and SMAD2/3 in the HC, HS and severe and very severe COPD groups. **c**. Mean fluorescence intensity (MFI) of BAMBI in monocyte-derived M2 macrophages in the HC, HS and COPD groups. **d**. The relative inducibility of M2 macrophages for Tregs in the HC, HS and COPD groups (fold change relative to the control). **e**. Ratio of CD86 + M1/CD163 + M2 macrophages in the HC, HS and COPD groups. **f**. The Mean fluorescence intensity (MFI) of BAMBI was negatively correlated with FEV1% in the COPD patients (*n* = 20). *P < 0.05; ^†^ *P* < 0.05, ^††^ *P* < 0.05, ^†††^ *P* < 0.05 (COPD group vs HC group); ^#^*P* < 0.05, ^###^*P* < 0.05 (COPD group vs HS group)

Since the expression levels of the TGF-β pathway and BAMBI in lung tissue, alveolar macrophages and CD4+ T cells in peripheral blood from COPD patients have been studied [24, 25, 28], here, we examined the relative expression of the TGF-β pathway and BAMBI in monocyte-derived macrophages in COPD.As shown in Fig. 3a, b and c, we observed a decreased expression of TGF-β1, TGF-βRI/II and p-SMAD2/3 and an increased expression of BAMBI in the severe and very severe groups (*n* = 6) compared with the HC and HS groups. (P < 0.05) Unfortunately, we did not find any significant differences in the TGF-β signalling pathway among mild to moderate COPD patients, HCs and HS subjects.

To investigate the ability of M2 macrophages to induce Tregs among the three populations, we analysed the proportion of Tregs after coculture and found a lower inducibility of Tregs in the COPD group than the other two groups (decreases of 31 and 55%), but there was no significant difference between the HC and HS groups (Fig. 3d).

In addition, the COPD group showed a higher ratio of CD86+/CD163+ (R = 1.20) than the HC group (R = 0.47) and the HS group (R = 0.81), and the difference was statistically significant (*P* < 0.01) (Fig. 3e).

Interestingly, as shown in Fig. 3f, BAMBI expression was negatively correlated with the forced expiratory volume in 1 second (FEV1%) in COPD patients, indicating that BAMBI may reflect the severity of COPD.

### Upregulation of BAMBI facilitated the conversion of M2 cells to M1 cells via the TGF-β/SMAD signalling pathway

**Fig. 4:**
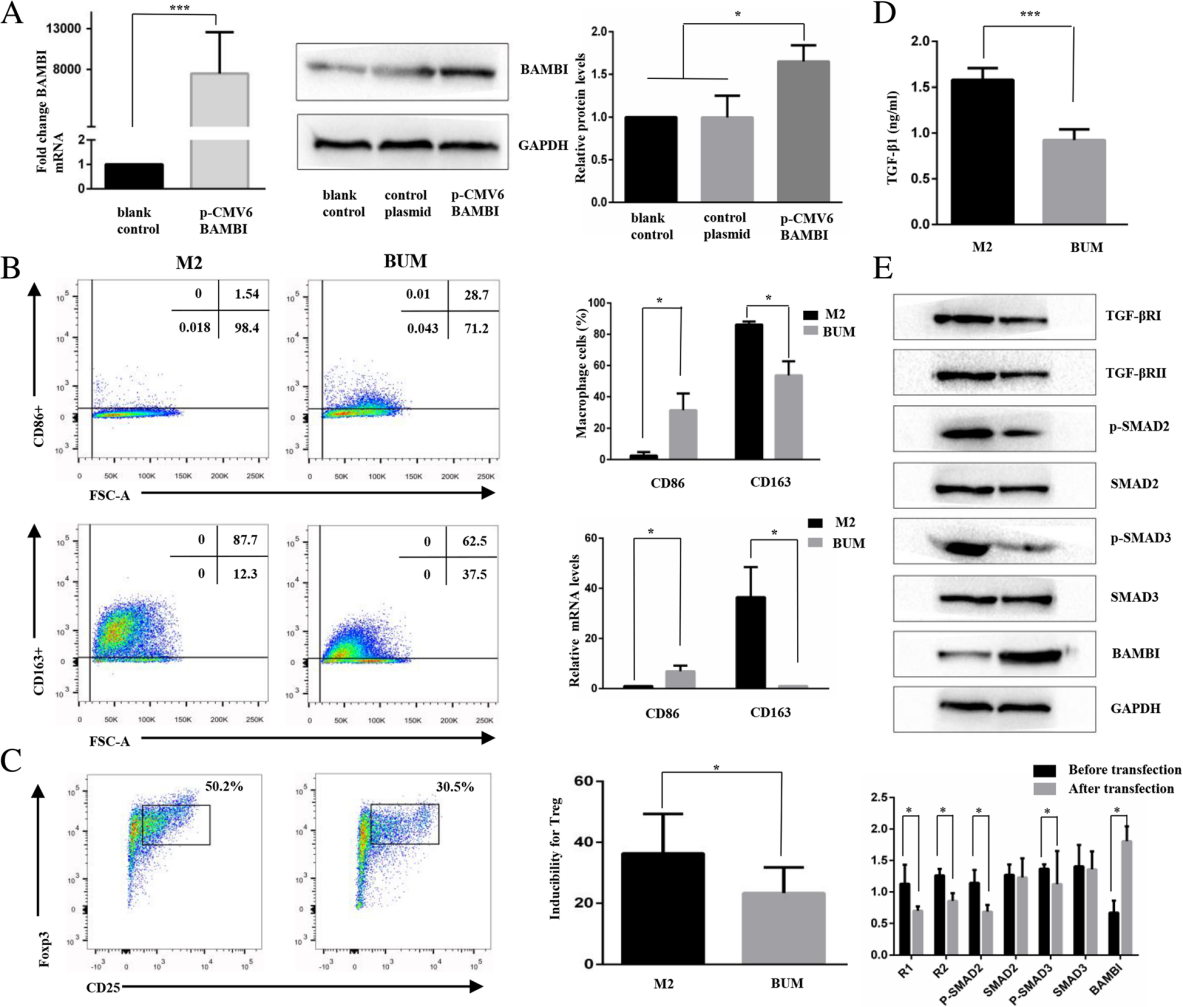
Overexpression of BAMBI facilitates the conversion of M2 to M1 cells through the TGF- β/SMAD signalling pathway. **a**. Transfection of pCMV-BAMBI into macrophages was validated by RT-PCR and WB. GAPDH was used as an endogenous control. **b**. Change in the phenotype characteristic between the BAMBI-upregulated macrophage (BUM) group and M2 macrophage group. **c**. The ability to induce the differentiation of CD4+ naïve T cells to Tregs in the BUM group and M2 cell group. **d**. Concentration of TGF-β1 in the BUM group and M2 cell group. **e**. The protein expression levels of TGF-βRI, TGF-βRII and p-SMAD2/3 in BUM group and M2 cell group. *P < 0.05, ***P < 0.001

To determine the role of BAMBI in the regulation of macrophages, we detected the characteristics of BAMBI-upregulated macrophages. First, we examined the transfection efficiency of pCMV-BAMBI in monocyte-derived M2 macrophages. As shown in Fig. 4a, the PCR and WB results indicated that BAMBI expression was significantly increased after transfection for 48 h.

Subsequently, we compared the phenotypes of M2 and BAMBI-upregulated macrophages. As shown in Fig. 4b, the RT-qPCR and flow cytometric results showed that BAMBI-upregulated macrophages expressed higher levels of CD86 and lower levels of CD163 than M2 macrophages (*P* < 0.05). These results indicate that BAMBI-upregulated macrophages had a phenotype similar to that of M1 cells. Then, we examined the ability of BAMBI-upregulated macrophages to induce regulatory T cells. As shown in Fig. 4c, the proportion of Tregs in the BUM group was decreased by 36% compared with that in the M2 group (*P* < 0.05). The results indicate that BAMBI-upregulated macrophages may express the characteristics of M1 cells.

As BAMBI has been previously identified as a pseudoreceptor of the TGF-β signalling pathway, to discern whether TGF-β/SMAD is involved in the mechanism of phenotypic and functional conversion, we examined TGF-β1 expression by ELISA and TGF-βRI, TGF-βRII and SMAD2/3 by WB analyses. The ELISA assays (Fig. 4d) showed that the concentration of TGF-β1 in the BUM group was decreased compared with that in the M2 cell group. As shown in Fig. 4e, the TGF-βRI and TGF-βRII expression levels were significantly decreased after the upregulation of BAMBI (*P* < 0.05). The expression levels of p-Smad2/3 also decreased (*P* < 0.05).

The results show that the TGF-β/SMAD signalling pathway is associated with the conversion of M2 cells to M1 cells both in phenotype and function, which weakened the anti-inflammatory environment.

## Discussion

In this study, we confirmed that M2 macrophages could induce the differentiation of Tregs through the TGF-β/SMAD signalling pathway. In addition, increased BAMBI expression and impaired TGF-β/SMAD signalling were found in M2 macrophages in severe and very severe COPD patients compared with those in the healthy smokers and healthy subjects. Furthermore, here, for the first time, we report that the upregulation of BAMBI could facilitate the conversion of M2 macrophages to M1 macrophages by negatively regulating the TGF-β/SMAD signalling pathway.

The supernatant of M2 cells was reported to induce the differentiation of Tregs [29]. However, Savage ND and his co-workers reported that M2 macrophages induced the differentiation of CD4+ T cells to Treg through a cell-cell mechanism [27]. Our results indicate that M2 macrophages rather than the supernatant of M2 cells could induce the differentiation of Treg. Different culture systems such as those with complex serum components for culturing macrophages, may account for these discrepancies. CD4 + CD25 + Tregs have also been reported to induce the differentiation of M2 macrophages partially through the IL-10 and TGF-β pathways in a mouse model [30], a previous study showed that human CD4 + CD25+ Tregs could induce the differentiation of M2a and M2c macrophages [31], in which way CD4 + CD25 + Tregs and M2 macrophages mutually promote each other through positive feedback, leading to a strong anti-inflammatory environment that is beneficial for the treatment of COPD. Some data also showed that Tregs exerted direct suppressive effects on the function of monocytes/macrophages [32]. In fact, the interaction between M2 macrophages and Tregs may be inhibited, although this mechanism has yet to be explored, especially in the inflammatory microenvironment of diseases such as COPD, and high expression of BAMBI might be one of the underlying mechanisms.

BAMBI has been reported to extensively participate in immunological diseases, such as pulmonary fibrosis [33], spinal cord injury [34], autoimmune arthritis [35]. In our previous report, BAMBI expression was upregulated in plasma from COPD patients [24]; here, we found that BAMBI expression in macrophages was significantly correlated with FEV1% in COPD patients, indicating that BAMBI may be a potential clinical biomarker for the diagnosis or treatment of COPD. Macrophages are highly plastic cells and M1 and M2 cells could be transformed into each other under certain circumstances [36]. Notably, increased BAMBI expression might convert M2 to M1 cells both in phenotype and function and, thus may lead to an enhanced inflammatory environment and reduced levels of Tregs. These changes can result in a reduced anti-inflammatory response, which may explain why COPD patients tend to be deteriorative and characterized by progressive airflow limitation.

Previous analyses have shown that the expression of the TGF-β/SMAD signalling pathway is elevated during the differentiation process through in vitro (RAW264.7) and in vivo studies (a mouse model) [16]. Increased TGF-β/Smad signalling molecules have also been reported in peripheral lung tissues from COPD patients [37]. However, several studies reached the same conclusion as we did [28, 38]. In our study, impaired macrophage function and TGF-β/Smad signalling pathway activation were observed in severe and very severe COPD patients, which may influence the immunologic function of regulatory T cells and in turn weaken anti-inflammatory reactions. This controversy may be explained by the use of different experimental models and different concentrations of CSE in the laboratory. As the TGF-β/Smad signalling pathway is widely involved in multiple immune reactions, the underlying disease in COPD patients might cause different immune states, leading to controversial results, and more rigorous studies are needed to confirm this concordance. In fact, decreased TGF-β1 and p-smad2/3 were detected after the upregulation of BAMBI [39]. Furthermore, the inhibition of BAMBI could activate TGF-β/SMAD signalling through the transfection of BAMBI-siRNA [40]. In this study, we confirmed that upregulate the expression levels of BAMBI may negatively regulate TGF-βR/Smad signalling pathway.

Cigarettes play an important role in oxidative stress, which is one of the important mechanisms responsible for the occurrence of COPD. Our study showed that stimulation with a specific concentration of CSE (0.02%) could promote the polarization of M0 macrophages to M2, and a similar result was reported in previous studies [16, 41]. In addition to monocyte-induced macrophage, macrophages from other sources have also been reported to be M2-polarized after CSE treatment [42–46]. The upregulation of M2-related genes and deactivation of M1-related genes have been reported in alveolar macrophages from healthy smokers [44, 47]. Eapen MS and his colleagues found that compared to HC and HS groups, the COPD group showed a dominant M2 phenotype in BAL, a mouse model and luminal areas, although the small airways showed an increase in M1 profiles [48]. Furthermore, the number of total macrophages in the HS and COPD groups was lower than that in the HC group [49]. Factors that cause oxidative stress such as INOS were found to be increased in COPD patients [50]. CSE-treated macrophages might also present weak phagocytosis [51], indicating that smoking might influence the antibacterial ability of macrophages, resulting in an uncontrolled pro-inflammatory environment.

Notably, due to the confusing definition of Tregs based on the phenotype, here, we used classical CD4 + CD25 + FOXP3+ cells to represent Tregs. Another limitation of this study is due to the relatively poor expression of BAMBI in macrophages, we did not carry out si-BAMBI studies along with pCMV-BAMBI to verify the induction process. Furthermore, we failed to find significant differences in the BAMBI and TGF-β/Smad signalling pathways between stable mild-to-moderate COPD patients and control subjects (including smokers with normal lung function); these issues should be assessed in further large-scale cohort studies. Despite its limitations, this study clearly indicates that BAMBI influences the function of macrophages in COPD, in which the TGF-β/SMAD signalling pathway plays a key role.

## Conclusions

Taken together, our results showed that M2 macrophages could induce the differentiation of Tregs via the TGF-β/SMAD signalling pathway. CSE was also demonstrated to convert M0 cells to M2 cells while increasing the expression of BAMBI. BAMBI was highly expressed in severe and very severe COPD patients compared with HC and HS groups, and the overexpression of BAMBI promoted M2 macrophages conversion to M1 macrophages through the TGF-β/Smad pathway. Furthermore, we discovered that increased BAMBI expression was negatively correlated with FEV1% in COPD patients, indicating that BAMBI may be a therapeutic target for COPD and improving our understanding of the pathogenesis of COPD.

## Abbreviations

BAMBI: bone morphogenetic protein and activin membrane-bound inhibitor;
BUM: BAMBI-upregulated macrophages
COPD: chronic obstructive pulmonary disease
CSE: cigarette smoke extract
FEV1: forced expiratory volume in one second
Foxp3: Forkhead Box P3
FVC: forced vital capacity
GM-CSF: Granulocyte-macrophage colony stimulating factor
M-CSF: Macrophage colony stimulating factor
PBMC: peripheral blood mononuclear cell
SMAD: small mothers against decapentaplegic
TGF-β: transforming growth factor beta induced protein
Tregs: regulatory T cells
